# Screening, brief intervention, and referral to treatment among homeless and marginally housed primary-care patients in Skid Row

**DOI:** 10.1186/1940-0640-7-S1-A58

**Published:** 2012-10-09

**Authors:** Lillian Gelberg, Ronald M Andersen, Lisa Arangua, Mani Vahidi, Blake Johnson, Vashti Becerra, Colleen Duro, Steve Shoptaw

**Affiliations:** 1 UCLA Department of Family Medicine & School of Public Health, Los Angeles, CA, USA; 2 UCLA Department of Psychology & Education, Los Angeles, CA, USA

## 

The University of California at Los Angeles Quit Using Drugs Intervention Trial (UCLA QUIT) tested a very brief primary-care–based screening and brief intervention (SBI) approach to reduce risky substance use and substance-related harm in safety-net clinics. The QUIT involves screening, very brief clinician advice (two to three minutes), and two telephone drug-use health education sessions versus usual care (control group) (n = 240 per condition). We present findings on unique recruitment issues in Skid Row, an east-central area of Los Angeles with a high population of homeless individuals. Between February 18 and April 28, 2011, previsit screening of adults in the clinic waiting room was conducted using a touch screen tablet PC. At-risk substance use was defined as casual, frequent, or heavy episodic use without the physiological or psychological manifestations of dependence (i.e., a score of 4 to 26 on the World Health Organization’s Alcohol, Smoking, and Substance Use Involvement Screening Test [ASSIST]). The focus of the study was on risky stimulant use, however, patients were screened for co-occurring alcohol, tobacco, and other drug use. A total of 920 patients were approached: 89% were ≥40 years old; 68% were male; and 62% were black. Of these, 706 were excluded prior to taking the ASSIST (reasons included being pregnant, presenting for a non-primary-care visit, being in substance use treatment, or refusal to participate). Of the 214 patients who completed the ASSIST, substance use rates based on scores were as follows: no/low risk, 11%; moderate risk, 42%; and dependence, 47%. Totals for each risk group, respectively, were as follows: tobacco (55, 101, 58), alcohol (62, 98, 54), cannabis (94, 77, 43), cocaine (89, 74, 51), amphetamines (145, 45, 23), inhalants (185, 20, 9), sedatives (143, 45, 26), hallucinogens (174, 30, 10), and opioids (130, 54, 30). Few patients qualified for the study because of substance use treatment or co-occurring alcohol or cannabis dependence. Key informants revealed that many of those approached received intermittent substance use treatment required by shelters. Enrollment criteria were relaxed to allow intermittent past-month substance use treatment or co-occurring alcohol or cannabis dependence. Enrollment rates increased several-fold. Our findings indicate SBIRT conducted in clinics with homeless and marginally housed populations must be tailored to their unique substance use and housing characteristics.

